# Genotyping and Phylogenetic Analysis of *Plasmodium vivax* Circumsporozoite Protein (*PvCSP*) Gene of Clinical Isolates in South-Eastern Iran

**Published:** 2020-05

**Authors:** Soudabeh ETEMADI, Mehdi NATEGHPOUR, Afsaneh MOTEVALLI HAGHI, Hamid ESLAMI, Mehdi MOHEBALI, Neda SETAYESH, Leila FARIVAR, Aref TEIMOURI

**Affiliations:** 1. Department of Medical Parasitology and Mycology, School of Public Health, Tehran University of Medical Sciences, Tehran, Iran; 2. Department of Medical Biotechnology, School of Advanced Technologies in Medicine, Tehran University of Medical Sciences, Tehran, Iran; 3. Department of Pharmaceutical Biotechnology, School of Pharmacy, Tehran University of Medical Sciences, Tehran, Iran; 4. Department of Parasitology and Mycology, School of Medicine, Zahedan University of Medical Sciences, Zahedan, Iran

**Keywords:** *Plasmodium vivax*, Circumsporozoite protein, Genotyping, Phylogenetic analysis, Iran

## Abstract

**Background:**

Circumsporozoite protein (CSP) is one of the most important surface sporozoite antigens in malaria, recently considered as a candidate for vaccination. Considering the importance of CSP, this study was conducted to investigate the polymorphism and genetic diversity of *Plasmodium vivax* Circumsporozoite Protein (*Pvcsp*) in the southeastern region of Iran during 2015–2016.

**Methods:**

To investigate polymorphism and genetic diversity, 20 blood samples were collected from patients with *P. vivax*, then DNA was extracted and amplified using partial sequence of CSP gene. Polymerase chain reaction (PCR) products were sequenced and compared to sequences from genomic databases using BLAST. Genetic evaluation and phylogenic analysis were performed using MEGA7 and DnaSP5 software’s on 38 sequences include 20 sequences of our study and 18 sequences of Gene Bank.

**Results:**

Eleven isolates were VK210 genotype and 9 isolates contained VK247. The result of variable segregation nucleotide site indicated that the differentiation of sequences in CSP were 25.67% in our 20 samples which are less than the 38 samples with a value of 26.67%. Comparing the ratio of dN/dS regions in the CSP gene indicates that the CSP varies more synonymously and amino acid has lower variation. Out of 38 samples, 35 unique haplotypes were identified based on 1042 nucleotide sequences in CSP, showing a variation percentage of 99.4%.

**Conclusion:**

The Tajima D analyses showed that CSP gene in *P. vivax* had a positive number in the total analyzed sequences, which means that the *P. vivax* mutations are in order to select positive evolution.

## Introduction

Malaria is one of the most common parasitic diseases in the tropical regions of the world, with more than 214 million new cases per year (1). According to WHO report total number of malaria cases verified in Iran was 1243 cases in 2015 that about 1158 cases were affected by *Plasmodium vivax*, which comprises 93% of the total number of malaria in Iran (2, 3). Sporozites are the first infective form in the life cycle of the parasite that enter human bloodstream and shortly after that enter into the hepatocytes of liver (4). The circumsporozoite protein (CSP) is the main surface protein of Plasmodium sporozoites attached specifically to molecules of hepatocytes membrane (5). Furthermore, CSP is a type of surface antigen that has multifunctional roles including; maturation of parasite and development of sporozoite, primary interactions between sporozoite and its host (mosquitoes and mammalian liver cells), and attacks the salivary glands of the mosquito and invasion of human liver cells (6).

Sequences that occur in CSP include a central repeat region that contains amino acid sequences and well-preserved regions I and II. Based on genetic diversity studies, two genotypes classified according to the central repeat regions including 9 amino acid repeats, are divided into VK210, VK247 and a third type has been identified in the central regions of the *P. semiovale* known as *P. vivax*-like (7,8).

Considering that this protein is a candidate for malaria vaccine, this study examined 20 human isolates of *P. vivax* to obtain genetic information using the CSP genetic marker and comparing these isolates with other isolates in the world and neighboring countries, and the dispersion of genotypes as well.

## Materials and Methods

### Collection of clinical isolates of Plasmodium vivax

Twenty *P. vivax* malaria blood samples were collected from patients who to referred district health centers of Chabahar, Sarbaz and Saravan districts in Sistan and Balouchestan Province (located in southeast of Iran) during 2015–2016.. The genomic DNA of the samples was extracted using High Pure PCR Template Preparation Kit (Roche Diagnostics, Germany) according to the manufacturer′s instructions and then were kept at −20 °C until molecular testing processes.

### Ethical approval

The current study was approved by the Ethics Committee of Tehran University of Medical Sciences with the associated code of ethics (number: IR.TUMS.VCR.REC.1395.1466). Furthermore, the informed consent from the participants was also obtained. A code was assigned for each of the patient and the data were kept totally confidential

### Duplication and sequencing of Pvcsp gene

**Fig. 1:**
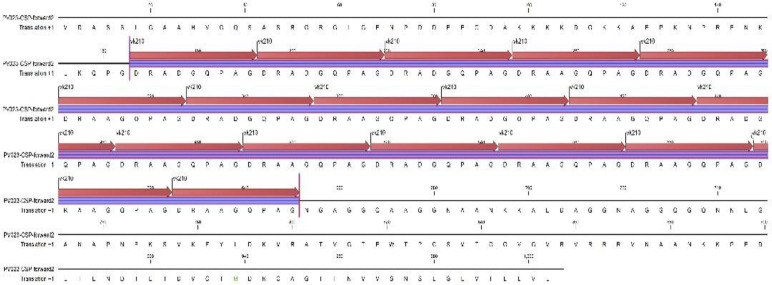
Schematic representation of the location of primers in the reference sequence

CSP gene was amplified using primer pairs of VCS1F 5′-ATG TAG ATC TGT CCA AGG CCA TAA A and VCS1R 5′-TAA TTG AAT AAT GCT AGG ACT AAC AAT ATG (Fig. 1).

Amplification was carried out using PCR-Ready Premix (Roche, Germany) in a 20-μl reaction include 5μL of template DNA, 1 μL of each primer, 10 μL of master mix (amplicon) content 0.4 unit tag polymerase and 0.125mM dntp with 0.8 mM Mgcl2 and 3 μL of distillated water. Reactions were as follow: 95 °C for 5 min, 30 cycle 60 °C for 1 min, 72 °C for 2 min, 94 °C for 1 min followed by 58 °C for 2 min and 72 °C for 5 min, respectively (9). Then, the product of PCR were analyzed in 1% agarose gels containing 2 μL of simple safe (EURx, Poland) and was visualized under UV. To determine genotypes of VK210, VK247 Using 1100 bp genome fragment the products were then purified using the Nucleo-Spin® Gel and PCR Clean-up purification kit according to manufacture instructions and were send to Bioneer Corporation in South Korea for sequencing.

### Data analysis

The nucleotide sequences were revised using Chromas version 2.4 and compared to sequences from genomic databases with BLAST. Accession numbers of 20 *P. vivax* isolates used in this study are recorded in Gen Bank as follows: MH802578- MH802579 MH802580-MH802581-MH802582-MH802583-MH802584-MH802585-MH802586 MH802587 and MH825646-MH825647-MH825648-MH825649-MH825650- MH825651 MH825652-MH825653-MH825654-MH825655.Genetic polymorphism practices including polymorphism site, genetic variation, drawing phylogenic tree and similarity percentage with the recorded genotypes were conducted through the software concerning the access to global sequences from the Gene Bank. Gene edit were performed using CLC genomic workbench 11 and APE software, and the drawing of the tree was performed using MEGA7 software and the evolution rate analysis with the DnaSP5 software (10). Phylogenic tree was drawn using Neighbor-Joining statistical method applying Kimura 2-parameter method for different strains of *P. vivax* parasite for CSP based on substitution changes in nucleic acid. Furthermore, evolutionary relationships of taxa the evolutionary history was derived exploitation the Neighbor-Joining methodology (11–13).

## Results

### Genotyping of Pvcsp in Iran

**Fig. 2:**
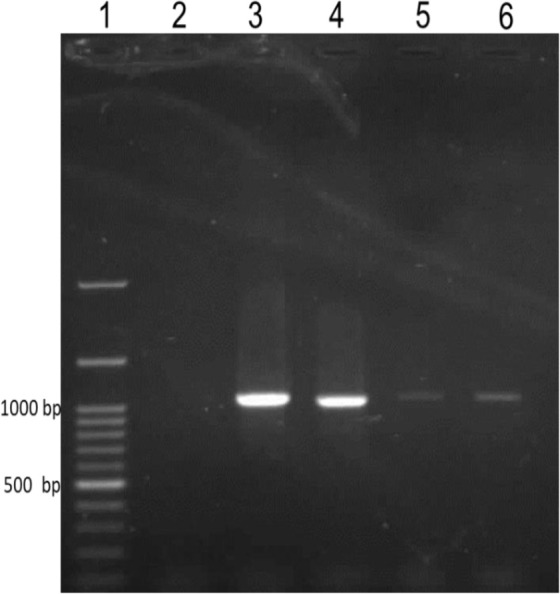
Illustration of 1100 bp bands of PCR products processed in 1% gel electrophoresis. Lane 1: 1 kb DNA ladder (SMOBIO, South Korea), Line 2: negative control, Line 3: positive control (Accession Number: HM535663.1), Line 4–6: samples of 1100 bp amplicons of *Pvcsp*

The amplification of the *Pvcsp* in the case of 20 isolates in the southeastern region of Iran showed the emergence of a band of 1100 bp (Fig. 2).

Among the isolates, those genotypes determined via repeated sequences based on the amino acid sequence, as well as considering that the repetitive region sequences of the GDRA [D / A] GQPA amino acids were related to the VK210 genotypes and the sequences of the repeated regions of the ANGAGNQPG amino acids were related to genotypes of VK247 and repeated sequences of APGANQE / GGAA amino acid were related to *P. vivax-*like genotype. Among the 20 studied isolates, 11 isolates were with genotype VK210 and 9 of them had genotype VK247, indicating that VK210 is still one of the dominant genotypes in the region.

### Comparison results of polymorphism of different Pvcsp sequences

Overall, 38 sequences include 20 sequences in our study and 18 sequences from Gene Bank were compared for the CSP gene of *P. vivax*. The result of variable segregation nucleotide site indicated that the differentiation of sequences in CSP was 25.67% in 20 samples of this study which is less than the 38 samples with a value of 26.67%. The comparison of informant change regions (parsimonious variables) against uninformed regions (Singleton variables) in the CSP shows that when the number of sequences increases from 20 to 38, parsimony regions that contain discriminating information do not have a significant change in the sequence. The number of complex codon regions in the read amino acids was 42 and increased with an increase in sample numbers. Comparing the ratio of dN/dS regions in the CSP indicates that the CSP varied more synonymously and amino acid varied less. Out of 1042 studied bases in CSP, 35 unique haplotypes were isolated from 38 samples, showing a percentage of 99.4% variation (Table 1).

**Table 1:** Information about the polymorphism of the informant area with the uninformed regions in the CSP gene sequences of *P. vivax* detached from the patient and compared with the Gene Bank sequences

***Genes***	No Seq^*^	No Nucleotide Bp^**^	***V (%)***	Singleton variable (%)^***^				Parsimony variable(%)^***^				***Synonymous/replacement change***			
				***2 variants***	***3 variants***	***4 variants***	***Total***	***2 variants***	***3 variants***	***4 variants***	***Total***	***C.C***	***dS (%)***	***dN (%)***	***dN / dS ratio***
Sequence	20	1036	266 (25.67	57	3	1	61 (23)	181	24	0	205 (77)	42	141	105	0.74
ALL	38	1042	278 (26.67	46	3	0	49 (17.62)	196	33	0	229 (82.37)	87	119	91	0.76

* Number of sequence that used in this study

** Total number of Nucleotides excluding gaps and/or missing data

*** Number of variant in non-informative or informative site. V: Variable (segregation) nucleotide site, Eta: Total number of mutations site. C.C: Total number of sites in other codons (complex codons) no analyzed because highly variable region. dS: Synonymous site, dN: Replacement site or no-synonymous site

Elevated nucleotide diversity per site π recorded from the 20 isolate π=0.116+−0.008 and haplotype diversity Hd of 1+ −0.016 and polymorphism=266, singleton=61, parsimony 205. Comparison of the mean of numbers of pairwise differences in total nucleotides (K) with the expected genetic variation in each sequence, on condition of the neutralization of evolution (Ɵ per seq), indicates that the K value is greater than Ɵ, that is, the expected mutation rate in *P. vivax* is positive. The Tajima D index indicates the type of mutation in terms of neutrality and this indicator shows a positive number in the total analyzed sequences, namely, the *P. vivax* mutations is in the positive selection of evolution (Table 2).

**Table 2:** CSP gene sequence polymorphism information in *P. vivax* detached from the patient and compared with gene bank sequences

***Genes***	***No. Seq.***	***No Nucleotide***	***S***	***Eta***	***K***	***π***	***Θ per site***	***Θ pre seq.***	***Tajima’s D***	***H***	***Hd***
Sequence	20	1036	266	295	109.58	0.113	0.077	74.977	1.921	20	1.00
All	38	1042	278	314	102.84	0.112	0.072	66.165	2.081^*^	35	0.994

* *P*<0.05. Total number of Nucleotides excluding gaps and/or missing data. S: Segregation site, Eta: Total number of mutations, K: Average number of pairwise nucleotide difference between pairs of sequences. Tajima’s D: the *D* test statistic proposed by Tajima, (Tajima, 1996). dS: synonymous site, dN: non-synonymous site, H: No. of haplotype, Hd: Haplotype diversity. π: nucleotide diversity, Θ: The amount of genetic variation

### Phylogenetic analysis

**Fig. 3:**
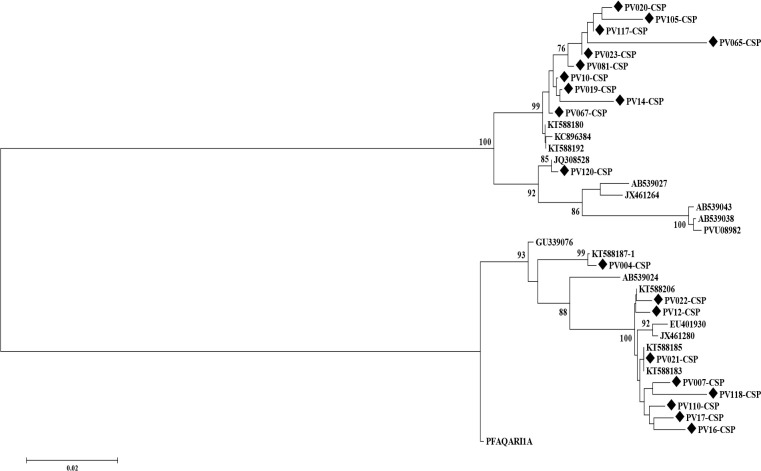
Phylogenic tree was drawn using Neighbor-Joining statistical method applying Kimura 2-parameter method for different strains of *P. vivax* parasite for CSP gene based on substitution changes in nucleic acid

The drawn tree topology shows that 2 clades with common ancestor for 38 sequences are observed, which causes the tree to be rooted. In the upper group (VK210), there are 11 cases of the studied sequences divided into two branches with common ancestors, the PV120-CSP sequence has a different genotyping of them sequences, and the sequence PV065-CSP is the oldest sequence in terms of the length of the evolution. Furthermore, in the bottom branch (VK247) PV004 is the strangest sequence in this category. In this topology, the accuracy of the tree is examined and, for example, the PV004 is different from other read sequences with this statistical model and it is detached (Fig. 3).

The best tree is represented with the sum of branch length=0.47091318. Moreover, the proportion of replicate trees area represent besides the branches (the bootstrap test is used here). An evolutionary distance has been used to draw a phylogenetic tree of scale. The rate of change between sites was modeled by the gamma distribution. The analysis included 38 nucleotide sequences, and in this genetic analysis, missing data and gaps were removed. Overall, 914 positions were available at the end of the data

## Discussion

*P. vivax* genetic recognition can be predicted by creating new phenotypes of parasites that play a key role in pathogenicity and drug resistance and the pattern of recurrence (due to hypnozoites), which ultimately has a positive effect on improving the health of individuals (14). Hypnozoites are intrinsic and inactive sporozoites (contain Circum Sporozoite Protein) in human liver parenchyma cells. Hypnozoites are seen in *P. vivax* infection, which results in a recurrence of the disease (relapses), the duration of the recurrence period varies in different strains of the parasite (there is frequent Relapse in tropical strains). Circum sporozoite protein (CSP) is important for the definition of population genetics, the phylogenic relationships of plasmodium species, pathogenesis (having polymorphism), and the transmission of infection from the vector of an anemone to a human being. Studies have also shown that CSP in different strains of Plasmodium Vivax is different (*Pvcsp*) (15–19).

In this study, *Pvcsp* was considered for a number of aims such as determining genotypes, to study the phylogenic structure, to find the percentage of nucleotide sequence similarity with sequences in the Gene Bank and polymorphism comparisons. *Pvcsp* gene encodes a protein that includes acid-amine repeated regions those bear epitopes of B cells as a immunogenic region that can cause immune response in humans and experimental animals (20, 21); In fact, studies of genetic variation in this gene can give more data about the function of its coding protein. The combination of the amino acids and the number of repetitive units indicates that the natural selection applied by the host system has shaped the features of these presentations (22). *Pvcsp* bears VK210, VK247 and *P. vivax* –like genotypes. In this study, out of the 20 examined isolates 11 had genotype of VK210 and 9 isolates included VK247 genotype, indicating that the result of this study is in agreement with those results obtained from the similar studies in different areas of the world (23). Moreover, genotype VK210 greatly has adapted in the world (24). Continually that the nucleotide diversity was 0.113% in the population of this study. Comparison of the ratio of the dN/dS in the CSP shows that the CSP has mostly synonymous variation and the amino acid is less variable. The repeated amino acid region shows that the peptide repeat structure is strongly preserved and the sequences have a common origin with two evolutionary trends. Our results are similar to those results obtained from of Columbia, Sri Lanka, and Iran (16, 23, 25), but not to those who reported changes less than the same name in the second domain (CR domain) (26). Out of 1042 bases in CSPs, 35 unique haplotypes were isolated from 38 samples, showing a percentage of variation of 99.4% and the expected mutation rate in the *Pvcsp* has been positive.

In tree topology using the Neighbor-Joining statistical method and the Kimura 2-parameter method for different strains of the Plasmodium vivax parasite for the CSP based on succession changes in nucleic acid, there are 2 clades with common ancestor for 38 sequences examined which causes the tree to be rooted. In the branches with common ancestors, the PV120-CSP sequence in the VK210 genotypes has a different genotyping, and the sequence PV065-CSP is the oldest sequence in terms of the length of the evolution. In addition, in the examined VK247 genotype sequence, PV004 is the strongest sequence. Although wide polymorphism was observed in the *Pfcsp* gene region for *P. falciparum* (which is the epitope of T cell), it was not seen for *Pvcsp* and it is unclear that changes in repeated domains can alter the specific immune response of the antibody.

In Iran, 80% of the isolates had the genotype VK210 and in total there are 25 different alleles and limited variation in the studied isolates occurred. The achievement of an effective vaccine based on the CSP alone or in compound by other antigens of different parasitic processes can be globally effective (16). Results of the extensive examination of *Pvcsp* polymorphism indicate that diversity in isolates is limited and this is consistent with other studies in the Southeastern Asia and Oceania and previous studies in Iran (27, 9).

Given the expected mutation rate in the gene *Pvcsp,* this study was in a positive direction. This could be the result of the emergence of resistant strains of *P. vivax* or for parasite escape from the host immune system and recurrence patterns in *P. vivax*.

## Conclusion

The Tajima D analyses showed that CSP gene in *P. vivax* had a positive number in the total analyzed sequences, which means that the *P. vivax* mutations are to select positive evolution. Furthermore, this study provides information for the malaria elimination program with the effect of transmission of *P. vivax* from neighboring countries in the region.

## Ethical considerations

Ethical issues (Including plagiarism, informed consent, misconduct, data fabrication and/or falsification, double publication and/or submission, redundancy, etc.) have been completely observed by the authors.
